# Home-based physical activity incentive and education program in subacute phase of stroke recovery (Ticaa’dom): study protocol for a randomized controlled trial

**DOI:** 10.1186/s13063-017-2410-9

**Published:** 2018-01-25

**Authors:** David Chaparro, Jean-Christophe Daviet, Benoit Borel, Benjamin Kammoun, Jean-Yves Salle, Achille Tchalla, Stéphane Mandigout

**Affiliations:** 1 Limoges University, HAVAE, EA 6310, F-87000 Limoges, France; 2 Department of Medicine and physical rehabilitation service, Limoges University Hospital, F- 87000 Limoges, France

**Keywords:** Stroke, Physical activity, Rehabilitation, Secondary prevention, Home-based exercise, RCT

## Abstract

**Background:**

Stroke causes functional decline, psychological disorders and cognitive impairments that affect activities of daily living and quality of life. Although physical activity (PA) is beneficial in stroke recovery, PA recommendations are rarely met after hospital discharge. There is presently no standard strategy for monitoring and inciting PA at home during the subacute phase of stroke recovery. The main aim of this study is to evaluate the effects of a home-based physical activity incentive and education program (Ticaa’dom) on functional capacity in subacute stroke patients.

**Methods:**

This study is a comparative prospective, observer-blinded, monocentric, parallel, randomized controlled clinical trial. This study will include 84 patients: 42 patients in the home-based physical activity incentive group (HB-PAI) and 42 in the control group (CG). The intervention group will follow the HB-PAI program over 6 months: their PA will be monitored with an accelerometer during the day at home while they record their subjective perception of PA on a chart; they will observe a weekly telephone call and a home visit every three weeks. The CG will receive traditional medical care over 12 months. The main study outcome will be the distance on a 6-minute walk test. Secondary outcomes will include measurements of lower limb strength, independence level, body composition, cardiac analysis, fatigue and depression state.

**Discussion:**

The results of this trial will demonstrate the value of implementing the Ticaa’dom program during the subacute phase of stroke recovery.

**Trial registration:**

ClinicalTrials.gov, NCT01822938. Registered on 25 March 2013.

**Electronic supplementary material:**

The online version of this article (doi:10.1186/s13063-017-2410-9) contains supplementary material, which is available to authorized users.

## Background

Strokes are experienced by almost 1.1 million people annually in Europe and cause approximately 1 in 19 casualties in the USA [1, 2]. Stroke patients commonly experience physical and cognitive impairments that affect activities of daily living (ADL), and contribute to sedentary lifestyles [3, 4]. Compared to their healthy peers, stroke survivors are less active, spend less time standing and walking [5], and engage in longer periods of uninterrupted sitting during the daytime [6]. However, physical activity (PA) and exercise are beneficial in all phases of stroke recovery [7, 8] and reportedly reduce cardiovascular risk factors [9, 10] while improving functional capacity and quality of life [11].

Systematic literature reviews reveal that stroke survivors receive an average of 2–6 days of PA monitoring after hospital discharge, and 3–6 months of home-based follow up [12–14]. The stroke population has a mean total energy expenditure (TEE) of around 321 kcal/day and a step count (SC) of 4355 steps/day, which is lower than the expected SC for people with chronic disability, i.e. 6500–8500 steps/day [13, 15]. Patients in the subacute phase of stroke recovery have significantly lower SC and spend more time in therapy and performing moderate activities compared to patients in chronic stroke recovery [16]. In this population, 30 minutes of cycle ergometer 3 days a week appears to be a sufficient amount of PA to increase peak oxygen uptake (VO_2_) and functional capacity and to improve the distance walked during a 6-minute walk test (6MWT).

Stroke patients often do not meet PA recommendations, i.e. at least 150 min a week of aerobic-moderate PA or 75 min a week of aerobic-vigorous PA [7]. Although there is no definite optimal strategy to encourage PA during subacute stroke recovery, potential approaches include home-based PA rehabilitation, structured rehabilitation exercises and secondary prevention programs [17–19].

On the subject of home-based PA in the subacute phase of stroke recovery, Studenski et al. [20] compared the motor control, balance, endurance and mobility of the upper and lower extremities following a 12-week cycle ergometer program. Compared to usual care, the intervention brought about better gains (*P* = 0.0056), in the form of improved scores in the Barthel Index (BI) and Balance Berg Scale (BBS), better peak aerobic capacity, improvements in the up-and-go test (TUG) and 6MWT, and better physical and social scores in the medical outcomes of the Short-Form 36-item (SF-36) questionnaire, better strength and improved upper extremity skills in subscales of the Stroke Impact Scale (SIS) and in quality of life (QOL) [20, 21].

To our knowledge, no study has yet investigated the effects of home-based PA incentive programs outside of standard and structured therapy during the subacute phase of stroke recovery. The investigated home-based PA incentive program (Ticaa´dom) could be a useful strategy to increase daily PA, to change sedentary lifestyle over time, and to establish PA monitoring after stroke. The primary aim of this planned study is to evaluate how a 6MWT is affected by the Ticaa´dom program over 12 months in the subacute phase of stroke recovery. We hypothesize that the PA incentive program will increase daily PA and independence among stroke patients. The secondary objective is to evaluate whether and how well patients keep practicing PA at 6 months after the end of the PA incentive program.

## Methods

### Ethics approval and considerations

All procedures will follow the recommendations of the institutional ethical committees per the Good Clinical Practice Guidelines of the Declaration of Helsinki. This project received authorization from the ethics and individual protection committee (IPC) (RCB: 2012-A01456-37) and is registered at http://ClinicalTrials.gov (NCT01822938). The Standard Protocol Items: Recommendations for Interventional Trials (SPIRIT) checklist is available as Additional file 1.

### Study design and settings

Ticaa’dom is a comparative prospective, observer-blinded, monocentric, parallel, randomized controlled clinical trial. The Neurological and Physical Medicine and Rehabilitation Services of Limoges University will conduct this study in the Limousin region of France.

**Fig. 1:**
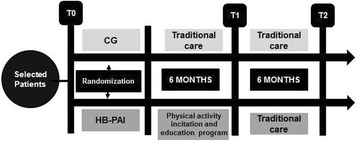
Study flow chart. *CG* control group, *HB-PAI* home-based physical activity incentive group

Ticaa’dom will also include a home-based 12-month post-discharge study conducted by professional PA therapists. The protocol will include two groups: the home-based physical activity incentive group (HB-PAI) and the control group (CG). The HB-PAI will include a physical activity incentive and education program for the first 6 months, followed by 6 months of traditional follow up. The CG will receive traditional follow up for 12 months (Fig. 1).

### Participants

Prior to hospital discharge, physicians will inform the patients of the purpose and methods of the study. The inclusion criteria will be the following: age ≥18 years; recovery from first ischemic or hemorrhagic stroke in all brain areas; stroke within <6 months; Functional Ambulation Classification score ≥2; patient monitoring by the post stroke interventional mobile team (HEMIPASS); and registration with the French social security system.

The exclusion criteria for both groups will be the following: stroke recurrence; disorders limiting gait skills before or after stroke; cognitive disorders impeding comprehension of physical activity education i.e. aphasia indicated by a Boston Diagnostic Aphasia Examination (BDAE) score <2; non-controlled hypertension; inability to complete questionnaires; cardiopulmonary pathology preventing effort completion; non-signature of written consent; involvement in other research; legal guardianship; and pregnancy.

### Intervention

#### Physical activity incentive and education program

The PA therapist will design and implement the PA incentive program for each participant. The program will include two phases: education and incentive. In the education phase, the therapist will explain the benefits of a regular physical activity practice. PA education will follow the recommendations of the French Society of Physical Medicine and Rehabilitation (SOFMER) and the French Neurovascular Society (SFNV) and will include educational diagnosis, selection of physical activities, and definition of goals [22].

The incentive phase will include monitoring, weekly telephone calls, and a home visit every 3 weeks for 6 months. A SenseWear armband accelerometer (BodyMedia, Pittsburgh, PA, USA) will be used to measure PA at home. The participant will wear the device during the daytime and remove it at night. The investigators will analyze the PA monitoring in three different categories: frequency (SC per week), PA duration, and PA intensity (TEE above 3 metabolic equivalents (METs)). The participant will complete a simple chart every day to measure subjective perception of PA for the physicians to compare the subjective and real amount and intensity of PA. The patients will receive a phone call once a week to encourage regular PA and to inquire about the PA measuring device. The PA therapist and HEMIPASS will perform a home visit every 3 weeks to discuss PA parameters – i.e. SC, TEE and physical activity duration – to incite regular practice, and to set objectives for the next visit.

### Control group

The CG will receive traditional follow up, including a medical appointment at 1 and 6 months after the hospital discharge. The CG will not monitor their PA and will not receive home visits or telephone calls. After inclusion, the PA therapist will give an explanation of PA recommendations and the benefits of an active lifestyle on stroke recurrence prevention.

The Ticaa’dom protocol is a strategy to monitor and incite PA at home in stroke survivors. In this context, ambulatory care in stroke rehabilitation – i.e. physiotherapy – will be authorized during the trial for both groups. In contrast, intensive after-discharge rehabilitation programs conducted by the hospital’s rehabilitation service or participation in other PA programs will not be allowed during the experiment. If hospital rehabilitation is mandatory, the participant will be excluded from the study.

### Outcomes

Outcome assessments will be performed after hospital discharge (T0), at 6 months (T1), and at 12 months (T2). The PA therapist will confirm the T1 and T2 medical appointments 3 weeks before the due date to avoid time bias. If the participant cannot attend the medical appointment, it will be delayed for a maximum of 3 days. The same practitioners, a medical doctor and a professional PA therapist will perform all assessments at the University Hospital of Limoges. The clinical examination, test and questionnaires will last around 90 min.

**Fig. 2:**
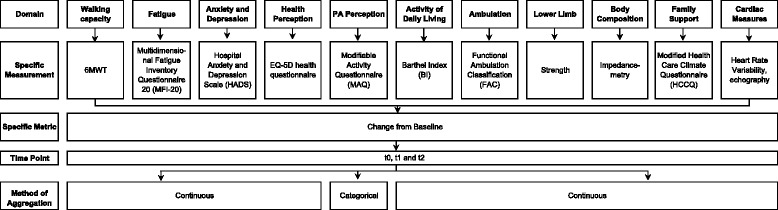
Outcome measures

The primary outcome will be the distance walked during the 6MWT, which is widely used in stroke studies [23, 24]. The 6MWT provides an objective measure of functional ability, endurance, and walking ability in people with physical impairments and stroke sequelae [25]. In addition, the 6MWT is a predictor of individual community walking activity, ambulation, and social integration in stroke survivors [26]. Patients will refrain from alcohol, tobacco, and caffeine consumption in the 24 hours before testing. The final score will be the distance walked during the 6MWT, and the rating of perceived exertion, assessed with the modified CR10 Borg scale from 0 to 10, will also be noted at the end of the test [27]. Secondary outcomes with the specific measurements are presented in Fig. 2.

The Multidimensional Fatigue Inventory questionnaire (MFI20) consists of 20 questions grouped in five dimensions and scored from 1 to 5. The Hospital Anxiety and Depression Scale (HADS) is a self-rated scale measuring anxiety and depression in hospital and in the community. The Euroqol-5D (EQ-5D) health questionnaire is a standardized instrument, which divides health status into five dimensions. The EQ-5 questionnaire is validated in hemiplegic and stroke patients [28, 29]. The Modifiable Activity Questionnaire (MAQ) evaluates the perception of PA through a listing of all activities done during the last 6 months. The Barthel index is a scale evaluating the dependence and independence of patients where a score of 0 represents total dependence and 100 represents complete independence. The Functional Ambulation Classification (FAC) classifies ambulation from 0 (nonfunctional ambulation) to 8 (independent ambulation). Lower limb strength will be measured three times per leg using the Dynatrac2 (Ysy medical). Finally, the heart rate variability test will be carried out in the resting and standing position according to the tilt-test protocol [30].

### Participant timeline

**Fig. 3:**
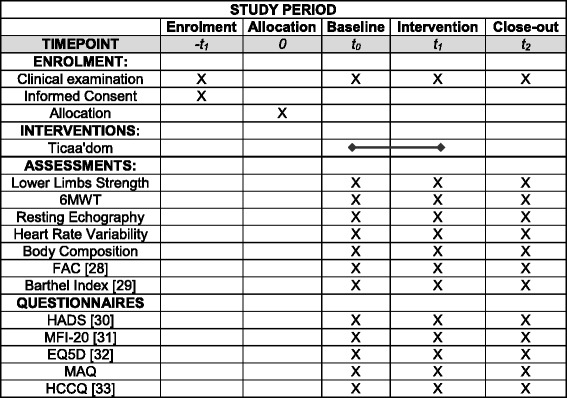
Participant timeline. *FAC* Functional Ambulation Classification, *HADS* Hospital Anxiety and Depression Scale, *6MWT* Six Minute Walk Test, *MFI* Multidimensional Fatigue Inventory questionnaire, *EQ-5D* Euroqol-5DHealth status questionnaire, *MAQ* Modifiable Activity questionnaire, *HCCQ* Modified Health Care Climate questionnaire

The Ticaa´dom protocol will start after the participant has signed the written informed consent form during the first clinical examination. Each patient will be evaluated in a specific order presented following the SPIRIT recommendations for interventional trials (Fig. 3).

### Sample size calculation

**Fig. 4:**
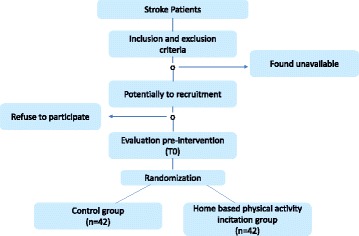
Patient flow chart

The Biostatistics unit of the Limoges University has calculated the sample size using the Nquery Advisor software (Version 6.1). The calculation is based on previous studies according to the protocol similarities in the home-based setting and protocol outcomes [31]. We hypothesized that the PA incentive program would improve the 6MWT distance by 30%. We have performed bilateral estimation with variance of 30%, alpha risk of 5% and power of 80%. The resulting required sample size was 36 subjects per group. We added 15% to ensure the statistical power of the study. As a result, the total sample will be 84 patients: 42 in the HB-PAI and 42 in the CG group (Fig. 4).

### Randomization and allocation

Eligible patients who agree to participate will be asked to give their written informed consent, and will then be randomized into the HB-PAI group or CG. We will randomize the patients at the first clinical examination. The randomization process will be ensured using a secure internet connection with access by individual user ID and password and encrypted data transmission to the randomization platform of the Clinical Research Unit of the Limoges University Hospital. Only the study methodologist will complete and keep the randomization list on a secure server. This randomization list will be balanced by blocks of variable size and will not include a stratification factor. All actions carried out on this platform and the identity of the person who made the changes will be automatically archived by audit trail procedure. As soon as a patient is randomized, an email will automatically be sent to all organizers of the study, i.e. the Clinical Research Assistant, Data Manager and Principal Investigator. No strategy was provided for preventing a potential imbalance between intervention and control group allocation.

### Blinding

The study will be partially blinded. Evaluation of all outcome criteria by a physical activity specialist dedicated to this matter will be performed blinded. A double-blind design is not possible for this study due to the methodology.

### Data collection, management, and analysis

A case report form (CRF) will be used to record all data, including physical activity variables and an explanation will be required for each missing piece of data. The PA therapist will collect, stock, and transcribe data clearly and legibly to the CRF.

The Biostatistics unit of the Limoges Hospital Center (CEBIMER) will create the database through the CAPTURE SYSTEM provided by CLINSIGHT Corporation. More specifically, they will adopt the “CS-DESIGNER” software to manage clinical databases using the ORACLE database.

The design and appearance of the database will be identical to the CRF. The data will be saved in real time and archived daily on a magnetic tape. The data history will be viewed through the AuditTrail system according to the computer security standards.

The Biostatistics unit will carry out consistency tests using the CAPTURE SYSTEM with a list of verification data registered on the QUERYS documentation. The investigators will check any correction made in the database twice to avoid bias before validating the database, preventing all further modifications of this version. The investigators will then export the database files in SAS format and send the tables back for statistical analysis. We will analyze the primary and secondary outcomes 6 months after final data monitoring and auditing.

### Statistical analysis

We will describe quantitative variables as mean ± standard deviation, and qualitative variables as numbers, percentages and 95% confidence intervals. We will present the study flow diagram and the patient characteristics for each group. The scientific council and the Biostatistics clinical research department will elaborate a detailed statistical analysis plan.

The analysts will compare the 6MWT distance between each group using either the matched Student’s *t* test or non-parametric Mann–Whitney test, depending on whether the data follow a normal distribution. They will also compare the qualitative variables using Pearson’s chi-squared test or Fisher’s exact test. We will compare the distributions of quantitative variables using Student’s *t* test or non-parametric Man-Whitney test.

The analysts will calculate the univariate Spearman coefficient for correlation between two quantitative variables. They will also compare data between T0, T1 and T2 in both groups using the Friedman test and Wilcoxon matched pairs test. Multivariate linear regression analysis may be performed, taking prognostic factors into account.

### Data monitoring and auditing

The Ticaa’dom intervention protocol does not present a major risk for participants. Thus, we will not establish an independent data monitoring committee (IDMC). A clinical research technician will always conduct data monitoring and auditing, and will be confirmed 3 weeks before the appointment. The auditing will ensure research quality, results validity and compliance with French laws and regulations.

### Harms

Pursuant to the French law regulating the Ticaa´dom protocol, the research team will report adverse events in the three following cases:

- Adverse event: any harm occurring to a person who is amenable to biomedical research, regardless of whether the event is related to the research (Article R.1123-39 of the Public Health Code)
- Serious adverse event: any adverse event that could cause the death or endanger the life of the participant, cause a significant or lasting disability or handicap, or lead to hospitalization or a congenital anomaly or malformation (article R.1123-39 of the Public Health Code)
- Unexpected adverse event: any adverse event where the cause, evolution, and/or severity is inconsistent with the information contained in the French protection committee applications (article R.1123-39 of the Public Health Code)

### Interruption and exclusion conditions

Authors will be empowered to remove any participant in the case of stroke relapse, serious adverse event, participant desire to withdraw, or development of any other condition impeding the participant’s independent movement. Patients who die will not be included in the analysis; patients who withdraw consent will be excluded from the study.

If the participant is absent at the evaluation or clinical examination, they will be excluded from the study, and will be followed up to determine the cause of the absence. We will take missing data into consideration by the last observation carried forward (LOCF) strategy. The PA therapist will document all withdrawals on the CRF along with the supplementary clinical information. A 15-day maximum period of pause will be tolerated.

## Discussion

The aim of the proposed study is to evaluate how a 6-month home-based physical activity incentive and education program affects functional capacity during the subacute phase of stroke recovery. The primary objective of the intervention is to encourage patients to meet PA recommendations and increase their 6MWT distance. The study is therefore designed to determine whether the home-based PA incentive program induces lifestyle changes and increases the amount of PA at medium-term and long-term follow up.

A previous study demonstrated that a home-based education program significantly reduced systolic blood pressure and body mass index, while it improved the distance the patient could walk after 12-month follow up [32]. However, the studied population included patients with transient ischemic attack, and their walking capacity and PA were measured subjectively (deliberate exercise walks). Therefore, to avoid these biases, our proposed study will only include stroke patients, and the protocol will provide objectively measured PA using a body-monitoring device.

Another previous randomized controlled trial showed that a home-based PA program had positive effects on the following: functional capacity, Barthel Index, physical and mental scores in the SF-36 questionnaire, and reduced risk of fracture after 12-month follow up [33]. Furthermore, a randomized controlled trial in a small sample of hemiplegic patients demonstrated that a home-based PA program was feasible, safe, and led to improved 6MWT distance and Timed Up-And-Go (TUG) test time [34]. Additional evidence is required on home-based PA in the subacute phase of stroke recovery.

The main limitation of the study will be the absence of PA monitoring in the control group. However, PA monitoring in the CG could bring about other forms of bias, such as the Hawthorne effect [35], i.e. the presence of a sensor could cause patients to change their lifestyles. Additionally, adherence to PA monitoring is reportedly low in stroke populations with low levels of balance, self-efficacy and walking endurance [36]. Despite this, remote PA monitoring is a common method used to quantify physical activity [14, 37]. Half of the studies in stroke patients have used accelerometers to measure walking or gait skills, and a third of them have used accelerometers to assess upper limb or arm movement [12].

Our home-based PA incentive program will not impose a standard or specific PA protocol. Incentive will be based on post-stroke PA recommendations and monitoring using a physical activity sensor (daytime), home visits (every 3 weeks), and telephones calls (every week). We will supervise the PA monitoring using this method during the PA incentive period (6 months). Overall, it is expected that the study will provide evidence on the benefits of the Ticaa’dom program in the subacute phase of stroke recovery. We hypothesize that the intervention will lead to improvements in physical, psychological, and sociological aspects related to daily PA.

### Trial status

The trial is currently in the recruitment phase. We have enrolled 50 out of our target 84 participants in the study.

## Additional file

Additional file 1: SPIRIT checklist. (DOC 120 kb)

## Abbreviations

6MWT: Six Minutes Walking Test
ADL: Activities of daily living
BDAE: Boston Diagnostic Aphasia Examination
CG: Control group
CRF: Case report form
EQ-5D: Euroqol-5D
FAC: Functional Ambulation Classification
HADS: Hospital Anxiety And Depression Scale
HB-PAI: Home-based physical incentive group
IPC: Individual Protection Committee
LOCF: last observation carried forward
MAQ: Modifiable Activity Questionnaire
MFI20: Multidimensional Fatigue Inventory questionnaire
PA: Physical activity
QOL: Quality of life
RCT: Randomized controlled trial
SC: Step count
SF-36: Short-form 36-item questionnaire
SFNV: French Neurovascular Society
SIS: Stroke Impact Scale
SOFMER: French Society of Physical Medicine and Rehabilitation
T0: Evaluation at hospital discharge
T1: Evaluation at 6 months
T2: Evaluation at 12 months
TEE: Total energy expenditure
TUG: Timed Up-And-Go
